# Comparative modular analysis of gene expression in vertebrate organs

**DOI:** 10.1186/1471-2164-13-124

**Published:** 2012-03-29

**Authors:** Barbara Piasecka, Zoltán Kutalik, Julien Roux, Sven Bergmann, Marc Robinson-Rechavi

**Affiliations:** 1 Department of Ecology and Evolution, University of Lausanne, Biophore, CH-1005 Lausanne, Switzerland; 2 Department of Medical Genetics, University of Lausanne, Rue de Bungon 27, CH-1015 Lausanne, Switzerland; 3 Swiss Institute of Bioinformatics, Lausanne, Switzerland

## Abstract

**Background:**

The degree of conservation of gene expression between homologous organs largely remains an open question. Several recent studies reported some evidence in favor of such conservation. Most studies compute organs' similarity across all orthologous genes, whereas the expression level of many genes are not informative about organ specificity.

**Results:**

Here, we use a modularization algorithm to overcome this limitation through the identification of inter-species co-modules of organs and genes. We identify such co-modules using mouse and human microarray expression data. They are functionally coherent both in terms of genes and of organs from both organisms. We show that a large proportion of genes belonging to the same co-module are orthologous between mouse and human. Moreover, their zebrafish orthologs also tend to be expressed in the corresponding homologous organs. Notable exceptions to the general pattern of conservation are the testis and the olfactory bulb. Interestingly, some co-modules consist of single organs, while others combine several functionally related organs. For instance, amygdala, cerebral cortex, hypothalamus and spinal cord form a clearly discernible unit of expression, both in mouse and human.

**Conclusions:**

Our study provides a new framework for comparative analysis which will be applicable also to other sets of large-scale phenotypic data collected across different species.

## Background

Specific over-expression of a gene in an organ is often taken to imply a function of the gene in that organ. If so, and if orthologous genes have conserved function, we would expect a slow rate of organ-specific expression evolution. Some early comparisons of microarray data between species suggested the opposite. The most studied data set in this regard is the GNF gene atlas of human and mouse organs [1,2]. Yanai, Graur and Ophir [3] used an early version of these data [1], and reported that the expression profiles of orthologous genes differed remarkably between two mammalian species. Moreover, comparing the expression profiles of 16 tissues (for both species), they found that human tissues were more similar to each other than to their corresponding mouse tissues. In contrast, Liao and Zhang [4], based on a more recent version of the data [2], and correcting for systematic error, found that human-mouse orthologous gene pairs had significantly lower expression divergence than random gene pairs. Additionally, they found that gene expression profiles of homologous tissues between species are more similar to each other than expression profiles of non-homologous tissues. Two recent studies [5,6] have confirmed that gene expression profiles of mouse and human homologous organs are indeed more similar than expression profiles between two different organs within a species, at least for the limited number of samples studied (immune system, heart and muscle, skin and gastrointestinal organs, liver and brain in [5]; kidney, liver, brain, spleen, skeletal muscle and lung in [6]).

In many of these studies the Pearson's correlation coefficient or Euclidean distance were used as estimators of gene expression conservation, either when calculating the distance between expression profiles of orthologous genes, or when clustering homologous organs from two species. These measures depend strongly on data normalization (Piasecka, Robinson-Rechavi and Bergmann, unpublished), and only capture global similarity across all samples. Specifically, none of these measures allows discovering between-species units of conservation, i.e., modules of organs and their specific genes that have remained largely unchanged since the speciation event. To facilitate gene expression studies, McCall et al. [6] have created a database of gene expression states in different conditions. It allows finding groups of co-expressed genes, but only for manually chosen conditions. Consequently, discovering modules of organs and their specific genes, requires an a priori guess about the potential groups of organs that express the same set of genes.

In this work, we take an alternative approach that automatically discovers such modules. We use the Ping-Pong Algorithm (that was originally developed for the unsupervised simultaneous modularization of gene expression and drug response data [7]) to co-analyze microarray gene expression data from mouse and human. Using the resulting co-modules, that contain genes and organs in which these genes are coherently expressed, we address several questions: 1) Are there any "natural" modules of mammalian organs, meaning groups of organs with very similar sets of co-expressed genes? 2) Which genes are module-specific? 3) Are these genes conserved between species?

## Results

### The ping-pong algorithm

**Figure 1:**
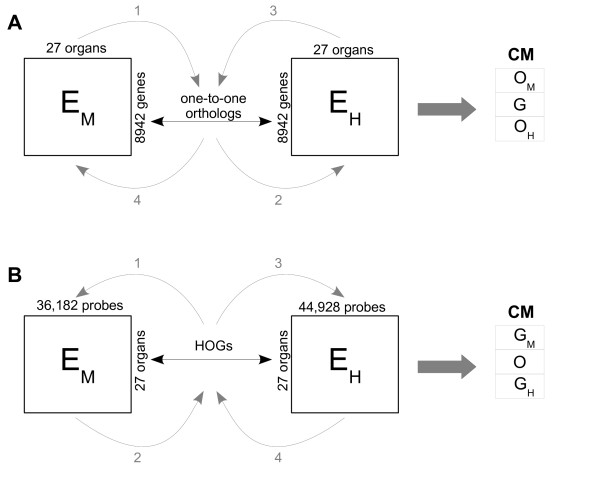
**Schematic representation of the Ping-Pong Algorithm**. (A) The PPA run for two data sets with orthologous genes on the common dimension. (B) The PPA run for two data sets with homologous organs on the common dimension. *E_H _*- human expression data, *E_M _*- mouse expression data, *O_M _*- mouse organs, *O_H _*- human organs, G - human and mouse one-to-one orthologs, *G_H _*- human genes, *G_M _*- mouse genes, O - homologous organs, CM - co-module.

The Ping-Pong Algorithm (PPA) [7] is an algorithm for the integrative analysis of two large-scale data sets sharing one dimension. When applied to gene expression data from two species, it identifies, simultaneously in both data sets, subsets of samples for which certain sets of genes are coherently overexpressed. We refer to the combined subsets of samples and genes as co-modules. The dimensions shared by our data sets are twofold: orthology relation between genes (Figure 1A) and organ homology (Figure 1B). First, we ran the PPA on the data sets matched through one-to-one orthologous gene pairs. Thus, the co-modules consisted of orthologous genes and the mouse and human organs in which these genes were overexpressed. Second, we ran the PPA on the data sets matched through homologous organ groups (HOGs) [8,9]. The resulting co-modules consisted of sets of homologous organs and (potentially different) sets of mouse and human genes with coherent overexpression in these organs. Each organ and gene received a score indicating their membership (if non-zero) and contribution to a given co-module. The further the score for a gene or organ is from zero, the stronger the association to the co-module.

Representing coherent features across both data sets in terms of co-modules reduces the complexity of the data and facilitates the study of its biological properties. There are only a few dozen co-modules to study, instead of thousands of genes. Moreover, the mean expression level of genes in a co-module is more robust than the expression measure for a single gene, as measurement noise tends to cancel out.

### Co-modules based on orthologous genes contain homologous organs

We applied the PPA to the mouse-human data sets matched through 8,942 one-to-one orthologous genes, containing the expression signal from 27 organs of both species. We ran the PPA starting from 10,000 different seeds consisting of random homologous organ groups. We obtained 25 distinct co-modules consisting of orthologous genes and the mouse and human organs where these genes were expressed.

Importantly, this analysis allowed us to recover the information about organ homology: co-modules contained mouse and human organs that are known to be homologous. The mouse organs which were grouped together with their human homolog were the following: lymph node, cerebellum, hypothalamus, tongue, testis, pancreas, liver and kidney. Moreover, we recovered information about functional groups of organs, which are conserved between mouse and human. In particular, we found a muscle co-module containing heart, skeletal muscle and tongue, a central nervous system (CNS) co-module with amygdala, and cerebral cortex, and an immune system co-module containing both lymph node and thymus. Genes and organs belonging to the same co-module were coherent in terms of functional annotation. For example, the muscle co-module was enriched in genes involved in glycolysis, the immune co-module in immune response, the testis co-module in sperm motility, and the liver co-module in catabolic processes (see additional file 1).

**Figure 2:**
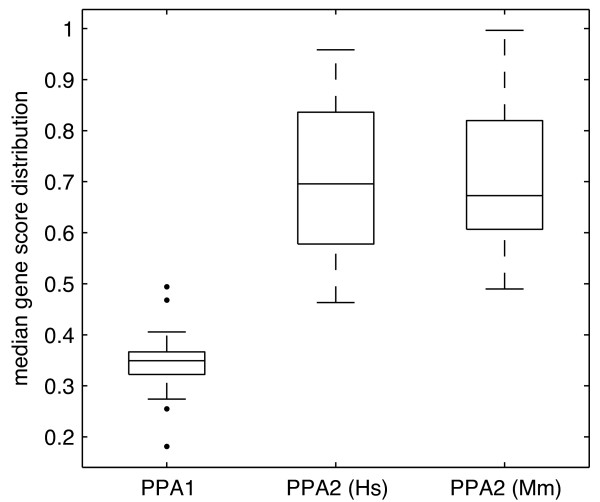
**Median gene score of co-modules from both Ping-Pong Algorithm runs**. (i) median gene scores for 25 co-modules detected in the PPA run on data matched through orthologous genes. (ii) median human gene scores for 98 co-modules detected in the PPA run on data matched through homologous organs. (iii) median mouse gene scores for 98 co-modules detected in the PPA run on data matched through homologous organs.

The median gene score for each co-module varied between 0.18 and 0.49 (Figure 2), which suggests that the contribution of individual genes to co-modules was rather weak.

### Co-modules based on homologous organs are organ- or system-specific

Above, we applied the PPA to the data sets matched through one-to-one orthologous genes. This recovered the information about organ homology and thus validated our approach, but limited it to one-to-one orthologous genes only. In a second step, in order to broaden the analysis, we applied the PPA to the data sets matched through 27 homologous organ groups. In contrast to the first run, here we used the expression signal coming from all 36,182 mouse probe sets and 44,928 human probe sets. We ran the PPA starting from 10,000 seeds consisting of random homologous organ groups. We obtained 98 distinct co-modules consisting of homologous organ groups and mouse and human probe sets carrying the signal specific for these HOGs. Next, the probe sets were mapped to their corresponding genes, and those which did not map unambiguously to a gene were excluded from further analysis.

**Figure 3:**
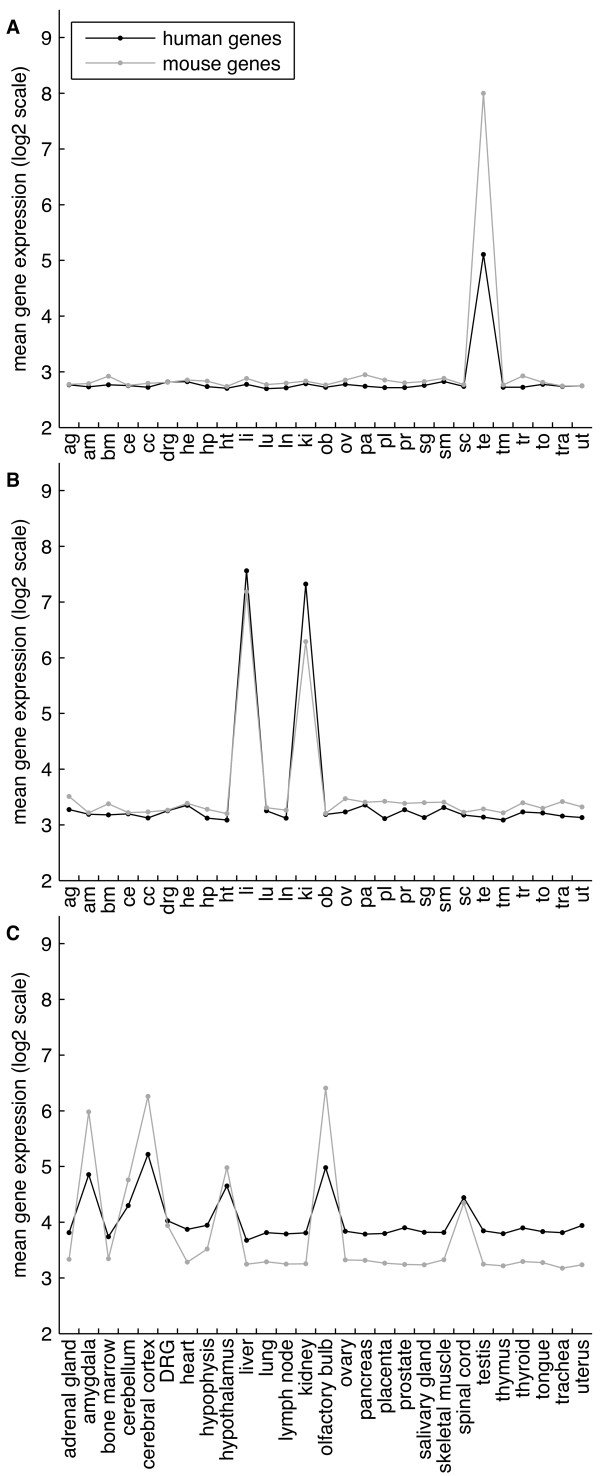
**Mean expression of genes belonging to three exemplary co-modules**. (A) testis-specific co-module; (B) immune system co-module containing lymph node and thymus; (C) co-module with two CNS organs assigned: cerebral cortex and olfactory bulb, but with an evidence for the gene expression also in amygdala, hypothalamus, and spinal cord.

First, for every single organ we detected a co-module containing this organ and its specific genes from mouse and human (Figure 3A), which confirms that organs are "natural" modules of gene expression in mammals. We refer to these co-modules as *organ-specific *co-modules. The median numbers of mouse and human genes assigned to these co-modules were 117 and 264.5, respectively.

Second, we confirmed and extended the discovery of co-modules containing several functionally related organs. We refer to them as *system-specific *co-modules. These notably include ovary and uterus; lung and trachea; lymph node and thymus; and liver and kidney (Figure 3B). The median numbers of mouse and human genes assigned to these co-modules were 257 and 281, respectively.

Third, the central nervous system (CNS) emerged as a particular case of a system-specific co-module. For instance, we found co-modules consisting of: amygdala, cerebellum and cerebral cortex; amygdala, hypothalamus and spinal cord; or cerebellum, hypothalamus, and spinal cord. After closer analysis of these co-modules we found that four central nervous system organs were connected more tightly than the others. These organs were: amygdala, cerebral cortex, hypothalamus and spinal cord. Whenever a co-module detected by the PPA contained one of these four CNS organs (e.g., cerebral cortex and olfactory bulb, Figure 3C), the genes from that co-module were also expressed in the three other CNS organs, although sometimes just below the threshold level that PPA used to add the organ into co-modules (see Methods). The median number of mouse and human genes assigned to these co-modules were 336 and 149, respectively.

The median gene score for each co-module varied from 0.46 to 0.96 for human, and 0.49 to 0.99 for mouse (Figure 2). The genes' contribution to co-modules was stronger than in the analysis with genes on the common dimension, which indicates that these co-modules are more reliable. This is probably due to the larger data sets used.

### Genes belonging to co-modules are enriched in functions relevant to the corresponding organs

Functional annotation analysis confirmed that genes belonging to each co-module were enriched in functions relevant to the respective organs, for both mouse and human. For example, the testis co-module was enriched in genes involved in spermatogenesis and sperm motility, the heart co-module in those involved in regulation of heart contraction, the lymph node co-module in those involved in immune response, and the nervous system co-modules were enriched in genes important during nervous system development (see additional file 2). This confirms the functional coherence of the organ- or system-specific co-modules detected.

### Organ-specific gene expression is often related to organ-specific hypomethylation of regulatory elements

**Figure 4:**
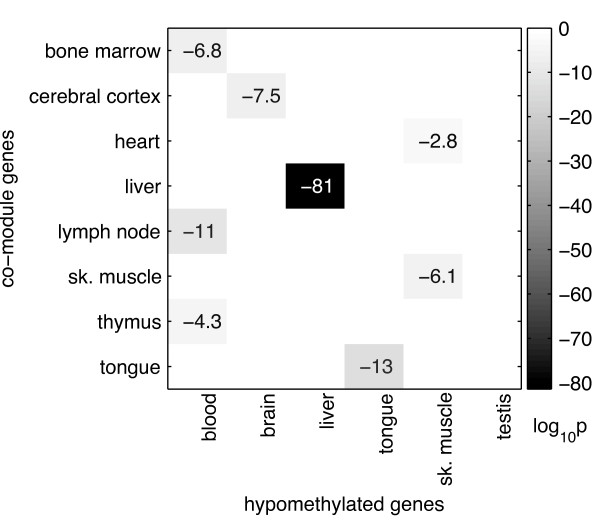
**Relation between organ-specific expression of genes and organ-specific hypomethylation of their regulatory regions**. For every organ-specific co-module we calculated the overlap between human genes belonging to the co-module and genes reported to be hypomethylated specifically in blood, brain, liver, tongue, skeletal muscle, and testis [10]. Only co-modules with significant overlap are presented on the heat map. The shade of grey corresponds to the corrected P-values of hypergeometric test in log10 scale.

Recently, Nagae et al. [10] reported a strong association between hypomethylated CpG-poor promoters and tissue-specific patterns of gene expression. We found a very significant overlap between our results and these of Nagae et al, for five out of six common tissues between both studies. For instance, genes that were hypomethylated in a brain-specific manner were over-represented in our cerebral cortex-specific co-module (*p *= 3.1 × 10^-8^), and genes hypomethylated specifically in the liver, were overrepresented in liver-specific co-module (*p *= 3.6 × 10^-82^). See Figure 4 for a summary of the results.

### Constraint on gene sequence is organ-specific

To check whether sequences of genes assigned to different co-modules evolve under different selective pressure, we computed their nonsynonymous to synonymous substitution ratios (*d_N_*/*d_S_*). For most co-modules the selective pressure did not differ from a random expectation (see Methods for test details). However, genes belonging to CNS-specific co-modules had significantly lower *d_N_*/*d_S_*, and genes from co-modules related to lymph node, liver, and testis had significantly higher *d_N_*/*d_S_*, than expected by chance (additional file 3).

### Genes' essentiality, duplicability, and age are weakly related to organ-specificity

Looking for other gene characteristics that may be related to different co-modules, we also studied: 1) gene essentiality, 2) gene duplicability, and 3) gene age (for details see additional file 4). First, we did not detect any significant relation between the co-modules and essentiality of the genes. Second, we found that CNS-related co-modules are significantly enriched in duplicated genes. Further studies are needed to investigate the causality of this relation. Third, we found that human genes from four co-modules and mouse genes from fourteen co-modules had an age distribution significantly different than expected. Importantly, only two co-modules were consistent in the age distribution for mouse and human genes, i.e., the tongue-trachea co-module showed an overrepresentation of young genes (Euteleostomi and later taxonomic levels), and the cerebellum-olfactory bulb co-module showed an overrepresentation of old genes (Bilateria). A few other CNS-related co-modules showed a similar age distribution, but only for mouse genes (figure S1 in additional file 4). In addition, we found that testis-related genes in mouse were enriched in genes from the Chordate level, and tongue-related genes were particularly young (Euteleostomi and later taxonomic levels). For human only we found that thymus-related genes were enriched in very old genes (Fungi/Metazoa). While these observations were significant in terms of statistics, they were not supported by consistent evidence from both mouse and human. This makes the interpretation of any relationship between gene age and co-modules difficult. Like for duplicability, we believe that further studies with more data will be necessary.

### Gene expression is conserved between mouse and human organs

**Figure 5:**
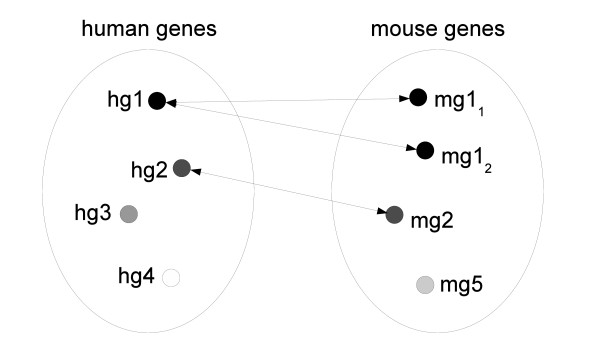
**Estimating the expression conservation rate (*γ*) of co-modules**. For every co-module we calculated the following numbers: *n_og _*- number of orthologous groups, $n_{fam_{h}}$ - number of all human gene families, $n_{fam_{m}}$ - number of all mouse gene families, and *γ *- the expression conservation rate. Here, *n_og _*= 2, $n_{fam_{h}}=4$, $n_{fam_{m}}=3$, $\gamma =n_{og}/min\left(n_{fam_{h}},n_{fam_{m}}\right)=2/3$.

**Figure 6:**
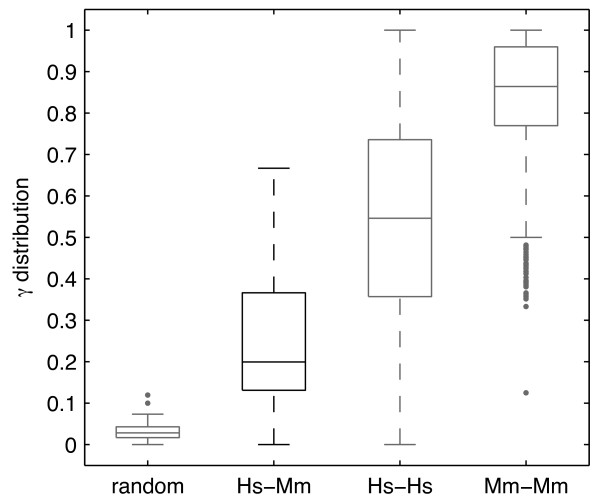
**Distribution of expression conservation rate (*γ*)**. Value of *γ *was estimated in four different cases: (i) for co-modules containing randomly paired human-mouse genes; (ii) for co-modules containing human-mouse orthologous genes; and for co-modules containing replicated human probe sets (iii) and replicated mouse probe sets (iv).

**Figure 7:**
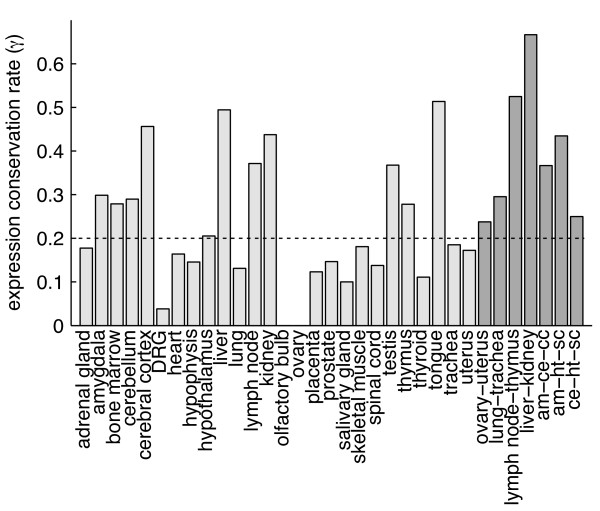
**Expression conservation rate (*γ*) for organ-specific and selected system-specific co-modules**. The median *γ *for all co-modules is marked with dotted line. Abbreviation for CNS-specific co-modules: am - amygdala, ce - cerebellum, cc - cerebral cortex, ht - hypothalamus, sc - spinal cord.

In order to study gene expression evolution between mouse and human, we calculated the rate of expression conservation (*γ*) for all co-modules resulting from the PPA run on data sets matched through homologous organs. We defined *γ *(eq. 1, Methods) as the ratio between the actual number of orthologous groups in a given co-module, and the maximal possible number of orthologous groups, i.e., the minimum of the number of human gene families and the number of mouse gene families present in this co-module (Figure 5). Thus the values of *γ *ranged from 0 to 1, with higher values indicating higher gene expression conservation in a given co-module. To assess if *γ *was significantly higher than expected by chance, we calculated it also for randomly paired mouse and human genes. The median *γ *for mouse-human orthologous genes was equal to 0.20, while for randomly paired genes the median *γ *was equal to 0.03 (Figure 6). Thus, the conservation of gene expression in mammals was significantly higher than expected by chance (*p *= 6.5 × 10^-6^, Mann-Whitney U test). *γ *values for all co-modules are shown in Figure 7 and in additional file 5. To determine the upper bound of the expression conservation rate that can be detected by our method with these data, we applied the PPA also to mouse-mouse and human-human data sets constructed by distributing the technical replicates from [2] into two disjoint sets. If these replicates had given identical expression profiles, we would observe *γ *= 1. However, due to experimental noise even using the replicate data one expects smaller values for *γ*. Indeed, this was the case for both comparisons, with a median *γ *of 0.86 for mouse replicates and a median *γ *of 0.55 for human replicates. Such low values of *γ *for data sets with identical underlying biological gene expression suggests that the values of *γ *which we obtained for human-mouse comparison probably underestimate the actual expression conservation.

### Gene expression is conserved between mammalian and fish organs

Given the conservation of expression between mouse and human organs, we asked if this is also true for more distant vertebrates. Using a modified version of the topGO R package [11] (Roux and Robinson-Rechavi, unpublished), we assessed organ expression enrichment for the zebrafish orthologs of genes, which belonged to the co-modules detected within the PPA run on data sets matched through organs, and were conserved between mouse and human. In other words, we measured in which zebrafish organs these orthologs were expressed more often than expected by chance. We found conservation of gene expression both for organ-specific co-modules, such as heart or liver, and for nervous system co-modules. For example, genes conserved in the co-module consisting of amygdala, cerebral cortex, hypothalamus, olfactory bulb and spinal cord in mammals, were found to be expressed in the following nervous system organs in zebrafish: retinal ganglion cell, trigeminal placode, cranial ganglion, and spinal cord in fishes. An exception to the general pattern of conservation was that the zebrafish orthologs of mammalian testis-specific genes seemed to be expressed in a wider variety of organs, including Kupffer's vesicle, the peripheral olfactory organ or the pronephric duct, but not including the zebrafish testis (see additional file 6).

## Discussion

Our methodology has allowed us to find "natural" modules of mammalian gene expression. In the first PPA run, with genes on the common dimension, we were able to recover the information of organ homology based only on orthologous genes expression patterns. In the second PPA run, with organs on the common dimension, we found organs grouped into homologous systems (between mouse and human), and their functional genes in both species; the latter were enriched in, but not limited to, orthologous genes.

According to our results the whole nervous system, and amygdala, cerebral cortex, hypothalamus and spinal cord in particular, forms a clearly discernible module both in mouse and human. Co-clustering of amygdala, hypothalamus and spinal cord was also reported in [4]. We found several other functionally related co-modules, for instance a co-module containing kidney and liver, a co-module related to the immune system (including lymph node and thymus), a female reproductive system co-module (ovary and uterus), or a respiratory system co-module (lung and trachea). A recent study of Brawand et al. [12] also showed that neural tissues (brain and cerebellum), and kidney and liver, form expression modules in amniotes. Grouping of some of the nervous system organs, was also reported in [5], but it was not possible to know exactly which CNS organs group together, as their annotation was simplified to "brain + nerve". The only other system reported in [5] combines heart and muscle. In their PCA results heart and muscle formed two distinguishable units, which were then grouped by the authors. Here, we found heart and muscle in a single co-module with the PPA run on data matched by orthologous genes, and in two separate co-modules with the PPA run on data matched by homologous organs. In the latter case the gene scores were higher (Figure 2), which suggests that heart and skeletal muscle, although similar, compose two distinct units of expression.

In addition to system-specific co-modules, we also found organ-specific co-modules. Thus with the PPA it is possible to simultaneously detect genes specific for a certain organ and genes shared between organs which form a system. On average, in mouse, there were less organ-specific genes than system-specific genes. No significant difference was found for human genes. All co-modules contained genes whose function was clearly related to the respective organs, justifying our notion of organ/system-specificity for the co-modules. This also confirms that an overexpressed gene has an important role in a given organ or organ system, in agreement with common expectations.

We explored the cause of organ-specific patterns of expression. One possible explanation was proposed by Nagae et al. in [10]. They discovered that genes with CpG-poor regulatory regions hypomethylated in an organ-specific manner tend to be expressed in an organ-specific manner. Indeed, for all but one of the organs that were included in their study we found that a significant fraction of the genes from our corresponding organ-specific co-module was hypomethylated. The only exceptions were testis-specific genes, for which we did not find evidence of hypomethylation in their promoter regions. However, genes that are specifically hypomethylated in testis tend to have CpG-rich promoters [10]. Thus, further work is needed to understand the regulation of testis-specific expression.

Our analysis of protein-coding gene sequences shows that the selection pressure on gene sequence is organ-specific. In particular, genes of CNS-related co-modules evolve slower in sequence, and genes from the co-modules related to lymph node, liver, and testis evolve faster, than expected by chance. These results are consistent with other reports that compared sequence evolutionary rate between human and chimpanzee [13], and between human and mouse [14]. A possible explanation for slower evolution of neural related genes was given by Drummond and Wilke [15]. These authors suggested that the structure and lifetime of tissues composed of neurons make them extremely sensitive to protein misfolding, and thus selection against protein sequence mutations is higher in these tissues. Possibly, the conserved protein sequence might be also related to the higher duplication rate of the genes expressed in CNS. However, this hypothesis needs to be addressed by a more specifically tailored study.

We found that co-module-specific genes are often orthologous between mammals. On average about 20% of the genes present in a given co-module had their orthologs in the same co-module. Note that the co-module-specific gene expression conservation rate (*γ*) from our analysis is rather underestimated, because of the noise present in the data. Even for human and mouse replicates only 55% and 86% of the genes present in a co-module had their replicate in the same co-module. The latter figures indicate that higher quality data (e.g., RNA-seq) are needed to improve our knowledge of gene expression evolution in mammals (e.g., [12], preferably with more organs).

Interestingly, we discovered two organ-specific co-modules with no detectable signs of expression conservation (*γ *= 0), i.e., the ovary-specific and the olfactory-specific co-module. The observed lack of expression conservation between mouse and human ovaries might simply be the effect of differences in sampling from two species: the mouse samples came from young, sexually mature individuals, whereas human samples were mostly taken from elderly people [2]. Ovary function varies strongly with age, independently of evolutionary conservation. For the olfactory bulb co-module such an explanation is less likely (even though olfactory sensitivity decreases with age). Rather, the absence of any detectable sign of expression conservation in this co-module suggests that different genes are involved in olfactory function in mouse and human. Indeed, it has been reported that the olfactory sense genes were shaped by different evolutionary processes in rodents and primates [16-18]. This shows that with the modular approach it is not only possible to discover "natural" modules of expression, but also to address questions about their evolutionary history since the divergence of two species.

To further study the extent of gene expression conservation, we contrasted the mammalian conserved genes with the expression data from zebrafish. We found that genes expressed in the brains of both mammals were also expressed in the brain of zebrafish. Similarly, genes expressed in mammalian heart or liver were found to be expressed also in their zebrafish homologs. This is a remarkable result indicating that indeed organ/system-specific gene expression evolution is rather slow. The exception was that zebrafish genes orthologous to mammalian testis-specific genes appear to be expressed in a wider variety of organs. This is consistent with previous reports of fast evolution of genes expressed in testis [12,14,19,20].

Our comparative study of homologous organs between mouse and human has several advantages, relative to previous approaches [3-6,21-23]. First, we analyzed a larger data set than most previous studies, with 27 homologous organs of mouse and human. Second, using the PPA instead of hierarchical clustering of organs, we were able to distinguish homologous modules at different levels of resolution - single organ or organ systems. Third, it is straightforward from our analysis to identify organ-specific or system-specific genes and to further analyze their features, while in most studies only the Pearson's correlation coefficient between organs is reported. Fourth, in all studies concerning the comparison of orthologous genes or homologous organs expression profiles, one had to decide how to represent gene expression values if a gene is targeted by more than one probe set. Because it is not possible to say which probe set most accurately measures the real expression level of a given gene, some arbitrary choice must be made (e.g., calculating the mean over all probe sets [22,24], picking a random probe set [4,23], taking the probe set with the highest expression level [14,21], or removing genes covered by multiple probe sets [3,25]). In the case of the PPA on data sets with organs on a common dimension all probe sets are used. Thus, if at least one of the multiple probe sets mapped to a gene carries an informative signal, the PPA can detect it and automatically find the group of similar probes representing other genes. This is impossible with any of the methods of probe sets pre-processing mentioned before. Notably, as many as 34.9% of human genes and 8.4% of mouse genes were mapped to multiple probe sets. And around half of the multiple probe sets mapped to a given gene were not together in the same co-module (48.6% of human genes and 52% of mouse genes had half or less probe sets together in the same co-module), which is a strong indication that these probe sets do not all correctly represent a gene, or possibly that they represent alternatively spliced forms, which code for different protein isoforms in different organs.

Two recent studies also applied modularization as a mean for cross-species comparative analysis of gene expression data. Yang and Su [26] used our Iterative Signature Algorithm (ISA) [27] (a precursor of the PPA) to identify and compare organ-related modules in human and mouse. Contrary to the PPA, the ISA discovers modules for a single species only. To conduct an inter-species study, Yang and Su compared modules from two independent ISA runs. They found fewer and smaller modules than we did with the PPA. This may have been a consequence of using only a single threshold for genes and organs. Importantly, they observed little cross-species overlap between the modules both in the organ and gene dimension. Consequently, they concluded that the content of modules in mouse and human diverged extensively. However, they found that modules with corresponding organs in mouse and human usually were enriched for genes of the same biological function. Brawand et al. [12] used the ISA to analyze RNA-seq data from six tissues and ten species, limited to one-to-one orthologous genes. This allowed the identification of several modules, which confirm the correspondence between organ-specific expression and functional annotation of genes. This study did not investigate the evolutionary conservation of organ-specific gene expression, and the detection of functional systems was limited by the few organs studied (i.e., brain and cerebellum, kidney and liver). On the other hand, using ten species allowed the detection of changes of expression in amniote evolution. These examples, and our analysis, illustrate the power of the modular approach to answer diverse questions in evolutionary biology.

## Conclusions

In conclusion, gene expression defines organ-specific or system-specific co-modules. These co-modules contain functionally related genes that are conserved between species. Thus there does exist a conserved modularity of gene expression in vertebrates, and it is related to anatomical modularity (i.e., organs).

## Methods

### Gene expression data

We used human and mouse gene expression data of Su et al. [2]. This study was performed on the Affymetrix HG-U133A array as well as the custom array GNF1H for human, and on the custom array GNF1M for mouse. In total, expression profiles from 79 human and 61 mouse organs were measured, with 44,928 probe sets for human and 36,182 probe sets for mouse. We only took into account organs belonging to the homologous organ groups (HOGs) defined in the Bgee database [28](see http://bgee.unil.ch/bgee/bgee?page=documentation#sectionHomologyRelationships). Using the mapping available in the Bgee database we could map 36 human organs and 30 mouse organs to 27 HOGs. See additional file 7 for the list of HOGs and their corresponding organs. Microarray data were normalized with the gcrma package [29] of Bioconductor [30].

Before we applied the PPA to the human-mouse data we merged human and mouse organs into 27 HOGs. For every probe set in each HOG the arithmetic mean of the gcrma normalized expression values was calculated (each HOGs was represented by at least 2 microarrays).

To study if it is possible to recover the information about organ homology based on the expression patterns of orthologous genes, we applied the PPA to the data sets consisting of a subset of 8942 one-to-one orthologous gene pairs (see Mapping Probe sets to Ensembl genes in Methods) and their expression patterns in 27 homologous organ groups in mouse and human. If a gene was matched by more than one probe set on the microarray, we randomly picked one probe set to represent that gene.

To study organ expression conservation between human and mouse we applied the PPA to the data consisting of expression values for 27 homologous organ groups, 44,928 probe sets for human and 36,182 probe sets for mouse. This time, the probe sets were mapped to their corresponding Ensembl genes after the PPA run.

To estimate the expected values of co-module expression conservation (*γ*, equation 1) when the gene pairs show conserved expression patterns we used replicated experiments as two different data sets, both for mouse and human. Therefore, for each probe set in mouse data and for each probe set in human data we had two vectors of values representing its expression over the organs. We applied the PPA to the data sets that contained 36 organs and 44,928 replicated probe sets for human and 30 organs and 36,182 replicated probe sets for mouse. We did not merge the organs into HOGs, because it was straightforward to pair the organs between replicated experiments.

### Mapping probe sets to Ensembl genes

To assign the probe sets to their corresponding mouse or human genes we used the mapping available in Bgee release 6, based on Ensembl release 55. We kept only probe sets which matched to a unique Ensembl gene. A total of 15,123 probe sets corresponding to 13,855 mouse genes, and 23,921 probe sets corresponding to 15,338 human genes, were taken into account in our analysis.

### Mouse-human orthologous genes

Homology information of mouse and human genes was retrieved from Ensembl release 55 [31], using BioMart [32]. A total of 10,321 pairs of mouse-human orthologous genes had expression information in the data sets we used (9,982 mouse genes and 9,883 human genes). One-to-one orthologous pairs account for 86.6% (8,942/10,321) of all pairs.

### Ping-pong algorithm

A detailed description of the algorithm in the general case is given in [7]. In this specific study, the algorithm starts with ten thousand candidate seeds consisting of randomly chosen homologous organ groups (HOGs), for both runs. Further steps are presented on Figure 1. Here, we only detail the PPA applied to the mouse-human data matched through HOGs: (step 1) the mouse expression data are used to identify the genes that exhibit similar expression in a given set of HOGs. (step 2): this set of genes is then used to refine the set of HOGs by excluding those which have an incoherent expression profile and adding others that behave similarly relative to genes. (step 3): in the next step the human expression data are used to find human genes that exhibit similar expression in a given set of organs. (step 4): similarly to step 2 the set of human genes is used to further refine the set of HOGs. Finally, this refined set of HOGs is used to look for mouse genes that are co-expressed in these HOGs (step 1). This procedure is reiterated until it converges to stable sets of HOGs and mouse and human genes (so-called co-modules). Every HOG and every mouse and human gene in a given co-module have a score assigned (between 0 and 1). The closest the HOG/gene score is to 1, the stronger the association between the HOG/gene and the rest of the co-module.

The PPA was applied to the mouse-human data sets twice. First, the two data sets shared the gene dimension. Second, the two data sets shared the organ dimension. The second experiment was coupled with the control experiment, which aimed to compare two matrices of replicated data within a species. The control experiment was done both on mouse-mouse and human-human data, with organs on the common dimension. We repeated this experiment ten times for each species. In every run the two replicates for each organ were randomly distributed between the two matrices and a thousand seeds consisting of random HOGs were created.

In every run of the PPA (both types) we used various thresholds for genes and organs, ranging from 2.5 to 6, and from 1 to 4.5, respectively. The thresholding is done by calculating the mean and standard deviation of the gene/organ scores vector and keeping only the elements that are *t *standard deviation above the mean, where *t *correspond to the value of the threshold. If the gene threshold is high, then the co-modules will have very similar genes. If it is low, then co-modules will be bigger, with less similar genes. The same applies to the organ threshold and the organs belonging to the co-modules (see http://www2.unil.ch/cbg/index.php?title=ISA_tutorial for detailed explanation).

### Post-processing of the PPA Results

The procedure described below was applied to the co-modules resulting from the PPA run on data matched through homologous organ groups. As we ran the PPA with different sets of thresholds, redundant modules were obtained. Before further analysis we eliminated this redundancy. For each pair of co-modules we calculated the correlation *c_h _*between human gene scores in the first and in the second co-module, and the correlation *c_m _*between mouse gene scores in the first and in the second co-module. If *c_h_*·*c_m _*> 0.8, which implies that the pair of co-modules had a very similar content for both species, the co-module with a higher sum of the two thresholds for human and mouse genes was kept, and the other co-module was disregarded. This procedure reduced the number of co-modules from 556 to 414. Next, we eliminated co-modules that had less than 10 probe sets assigned for at least one species. This procedure reduced the number of co-modules further, to 231. Still, many sets of organs were represented by several overlapping co-modules. Consider two co-modules containing *H*_1 _and *H*_2 _sets of human genes, respectively. We say that two modules have fully overlapping sets of human genes *H*_1 _and *H*_2_, if either *H*_1 _⊆ *H*_2 _*or H*_2 _⊆ *H*_1_. For each set of co-modules with fully overlapping sets of human genes the biggest co-module was chosen for the further analysis, and the rest were disregarded. The size of a co-module was defined as the minimum of the two values: 1) the number of human genes in a co-module and 2) the number of mouse genes in a co-module. After this final step, there were 98 co-modules used in further analysis.

In order to assess the rate of gene expression conservation we used only orthologous gene pairs with corresponding probe sets present on both the human and mouse microarrays. The rate of the expression conservation in a co-module was calculated as

(1) $$\gamma =\frac{n_{og}}{min\left(n_{fam_{h}},n_{fam_{m}}\right)},$$

where *n_og _*is the number of orthologous groups in a given co-module, $n_{fam_{h}}$ is the number of human gene families in a given co-module for which ortholog(s) are present on the mouse microarray (but not necessarily in the same co-module) and $n_{fam_{m}}$ is the number of mouse gene families in a given co-module for which ortholog(s) are present on the human microarray (but not necessarily in the same co-module) (Figure 5). The same procedure was applied to calculate the *γ *for co-modules from mouse-mouse and human-human comparison, with *n_og _*being the number of probe sets present in replicates in a given co-module, and $n_{fam_{h}}$, and $n_{fam_{m}}$ being the total number of probe sets from the first and second experiment present in a given co-module.

To verify if the results of our analysis were different than expected by chance we created lists of random pairs of mouse-human genes. This was done ten times by reshuffling the list of 10,321 mouse-human orthologous pairs, in a way that kept the same number of one-to-one and many-to-many gene pairs. For every co-module and every list of random gene pairs we recalculated the *γ*. Finally, for every co-module the mean *γ *was calculated.

### Enrichment analysis of hypomethylated regulatory regions

To determine if hypomethylated regions are over-represented in genes belonging to organ-specific co-modules we used data from the work of Nagae et al. [10]. They provided the lists of genes specifically hypomethylated in: brain, tongue, liver, blood, skeletal muscle, and testis. We used these sets of tissue-specific hypomethylated genes, and intersected them with the genes from our organ-specific co-modules. We performed the hypergeometric test to verify if the genes reported in [10] were overrepresented in any of our co-modules. To correct for multiple testing we applied the Bonferroni correction.

### Gene sequence analysis

The one-to-one orthology relationship between mouse and human genes, and the values of *d_N _*(rate of nonsynonymous substitution per codon) and *d_S _*(rate of synonymous substitution per codon) were retrieved from Ensembl version 55 [31], using BioMart [32]. We used the set of 12,248 human genes with *d_N_, d_S_*, and microarray expression data. To assess whether the genes belonging to a given co-module have *d_N_*/*d_S _*ratios significantly different than expected by chance, we performed a Wilcoxon rank sum test comparing the median *d_N_*/*d_S _*from a co-module to the median *d_N_*/*d_S _*for all human genes. After the Bonferroni correction the significance level was set at *p *= 0.0005. We repeated the same procedure for 10,540 mouse genes.

### GO enrichment analysis

Gene ontology (GO) association for all genes mapped to mouse and human probe sets were downloaded from Ensembl release 55, using BioMart. GO enrichment was tested by Fisher's exact test, using the Bioconductor package topGO [11] version 1.12.0. The reference set consisted of all Ensembl genes mapped to probe sets of the microarray used. The "elim" algorithm of topGO was used to eliminate the (tree-like) hierarchical dependency of the GO terms. To correct for multiple testing (98 co-modules tested) the Bonferroni correction was applied. For every co-module only GO categories with corrected P-value lower than 0.05 were reported.

### Zebrafish-mouse orthologous genes

Homology information of zebrafish and mouse genes was retrieved from Ensembl release 55 [31], using BioMart [32]. Only mouse genes with expression conserved in mouse-human co-modules were used to find their zebrafish orthologs. A total of 1,892 pairs of zebrafish-mouse orthologous genes was found (1,560 zebrafish genes and 1,026 mouse genes).

### Organ enrichment analysis

Associations of zebrafish genes to anatomical ontologies were downloaded from the Bgee database, release 6. Association between genes and organs was based on expression patterns detected in *in situ *hybridization experiments (see Bgee documentation at http://bgee.unil.ch/bgee/bgee?page=documentation for more information). Enrichment of expression in organs was tested using a modified version of the topGO package [11] (Roux and Robinson-Rechavi, unpublished). To correct for multiple testing (82 co-modules tested) the Bonferroni correction was applied. For every co-module only zebrafish organs with corrected P-value lower than 0.05 were reported.

## Competing interests

The authors declare that they have no competing interests.

## Authors' contributions

BP, SB and MRR contributed to the research design. BP and JR gathered the data. ZK and JR contributed analysis tools. BP performed the analysis and wrote the original manuscript. ZK, JR, SB and MRR provided critical comments about the statistical analyses and revised thoroughly the manuscript. All authors read and approved the final manuscript.

## Supplementary Material

Additional file 1 **The list of co-modules obtained with the PPA run on data matched by orthologous genes**. For every co-module we show number of genes, list of human and mouse organs assigned to the co-module, and the list of enriched GO categories for genes belonging to these co-modules. Click here for file

Additional file 2 **The list of co-modules obtained with the PPA run on data matched by homologous organs**. For every co-module we show list of homologous organs, number of human and mouse genes assigned to the co-module, and the lists of enriched GO categories for human and mouse genes belonging to these co-modules. Click here for file

Additional file 3 **The list of co-modules obtained with the PPA run on data matched by homologous organs**. For every co-module we show the median *d_N_*/*d_S _*value for mouse and human genes separately. We also show a p-value of Wilcoxon signed rank test. In bold black wemarked modules with median *d_N_*/*d_S _*significantly lower than global median, and in bold red we marked co-modules with median *d_N_*/*d_S _*significantly higher than global median. Click here for file

Additional file 4 **Analysis of the relationship between the co-modules and genes' age, duplicability, or essentiality**. Click here for file

Additional file 5 **Expression conservation rate (*γ*) for other system-specific co-modules**. The median *γ *for all co-modules is marked with dotted line. Abbreviations for organ names: ag - adrenal gland; am - amygdala; bm - bone marrow; ce - cerebellum; cc - cerebral cortex; drg - dorsal root ganglion; he - heart; hp - hypophysis; ht - hypothalamus; li - liver; lu - lung; ln - lymph node; ki - kidney; ob - olfactory bulb; ov - ovary; pl - placenta; pr - prostate; sc - spinal cord; te - testis; tm - thymus; to -tongue; tra - trachea; ut - uterus. Click here for file

Additional file 6 **The list of co-modules obtained with the PPA run on data matched by homologous organs**. For every co-module we show list of homologous organs assigned to the co-module and the list of the zebrafish organs enriched in expression of genes orthologous to mouse genes assigned to the co-module. Click here for file

Additional file 7 **The list of homologous organ groups and their corresponding sample names in human and mouse expression data sets**. Click here for file
