# Supplementary material for: Comparative modular analysis of gene expression in vertebrate organs
Source: BMC Genomics. 2012 Mar 29;13:124. doi: 10.1186/1471-2164-13-124 (PMC3359279; doi:10.1186/1471-2164-13-124)
Supplement: Additional file 4 — Analysis of the relationship between the co-modules and genes' age, duplicability, or essentiality. [file 1471-2164-13-124-S4.PDF]

Supplementary Materials:
Comparative modular analysis of gene expression in vertebrate organs

Barbara Piasecka[1,2,3], Zoltán Kutalik[2,3], Julien Roux[1,3], Sven Bergmann[2,3†] and Marc Robinson-Rechavi[*1,3†]

[1]Department of Ecology and Evolution, University of Lausanne, Biophore, CH-1005 Lausanne, Switzerland
[2]Department of Medical Genetics, University of Lausanne, Rue de Bungon 27, CH-1015 Lausanne, Switzerland
[3]Swiss Institute of Bioinformatics, Lausanne, Switzerland

Email: Barbara Piasecka - Barbara.Piasecka@unil.ch; Zoltán Kutalik - Zoltan.Kutalik@unil.ch; Julien Roux - Julien.Roux@unil.ch; Sven Bergmann - Sven.Bergmann@unil.ch; Marc Robinson-Rechavi*- Marc.Robinson-Rechavi@unil.ch;

*Corresponding author
†Contributed equally

Analysis of essentiality of genes

To study the essentiality of mouse genes belonging to different co-modules we retrieved data on mouse mutants from the Mouse Genome Database (ftp://ftp.informatics.jax.org/pub/reports/MPK_Ensembl_Pheno.rpt). We extracted from the file MRK_Ensembl_Pheno.rpt all genotypes with assigned phenotype, and their mapping to Ensembl genes. For every co-module we considered all genes with any phenotype assigned and calculated the number of genes exhibiting the mortality/aging phenotype. We performed the hypergeometric test ($\alpha = 0.05$) to verify if the essential genes were overrepresented in any of our co-modules. To correct for multiple testing we applied the Bonferroni correction. We did not detect any significant relationship between the co-modules and the mouse essential genes.

Analysis of duplicability of genes

We studied the enrichment of co-modules in genes that underwent duplication in their evolutionary history. We retrieved the paralogous information of mouse and human genes from Ensembl release 55, using BioMart. For every co-module we calculated the number of duplicated genes. We performed the hypergeometric test ($\alpha = 0.05$) to verify if the duplicated genes were overrepresented in any of our co-modules. To correct for multiple testing we applied the Bonferroni correction. We found that among the co-modules significantly

enriched in the content of duplicated genes, the CNS co-modules form a majority (28/40 for human, and 31/47 for mouse). This suggests a relation between gene duplication and expression in the central nervous system. Also placenta, uterus, lymph node, and lung seem to be enriched in duplicated genes (table S1). Understanding the causal relation of gene duplicability and organ function was beyond the scope of this paper, and it still requires further studies.

Analysis of age of genes

To study the age of genes belonging to different co-modules we dated the genes by their first appearance in the phylogeny. This consisted of retrieving the age of the oldest node of their Gene tree in Ensembl release 55. For every co-module we calculated the age distribution of its genes. We performed chi-square goodness of fit test to compare the observed and expected distributions of age classes in the co-modules. Expected distribution was calculated for every co-module separately with a correction for d_N/d_S values of the genes. This correction was necessary because faster evolving genes are often assigned a younger taxonomic level than slower evolving genes. This is due to difficulties in properly detecting two genes as orthologs when they have fast evolving sequences. For example, in a lymph node-specific co-module genes have high d_N/d_S values and thus could be more often considered as young. Consequently, one should not compare the age distribution of a co-module to the age distribution of all genes.

In order to overcome this problem, we first sorted all genes according to their d_N/d_S values and divided them into four equal-sized bins. We then calculated the age distribution within every bin separately. Next for each co-module, we calculated the expected number of genes of different age as a weighted sum of the age distributions of all four d_N/d_S bins. The four weights were calculated as the number of genes from the co-module that belonged to corresponding d_N/d_S bins. Finally, the expected number and the observed number of genes of different age were compared for each co-module. To correct for multiple testing we applied the Bonferroni correction.

For vast majority of the co-modules we did not detect any significant difference in the age distribution ($\alpha = 0.05$). There were only four co-modules for human and fourteen co-modules for mouse for which we detected significant age variation (see figure S1). As discussed in the main text, even though these observations were statistically significant, their interpretation remains difficult. Further studies will be necessary to explain the relation between organ-specific gene expression and its age.

Figure S1 - Age profiles of co-modules with statistically significant age variation

y-axis corresponds to the difference between observed and expected frequency for a given taxonomic level. FM - Fungi/Metazoa, B - Bilateria, C - Chordata, E+ - Euteleostomi and later taxonomic levels. am - amygdala; ce - cerebellum; cc - cerebral cortex; ht - hypothalamus; li - liver; lu - lung; ln - lymph node; ki - kidney; ob - olfactory bulb; pl - placenta; sc - spinal cord; te - testis; tm - thymus; to - tongue; tra - trachea.

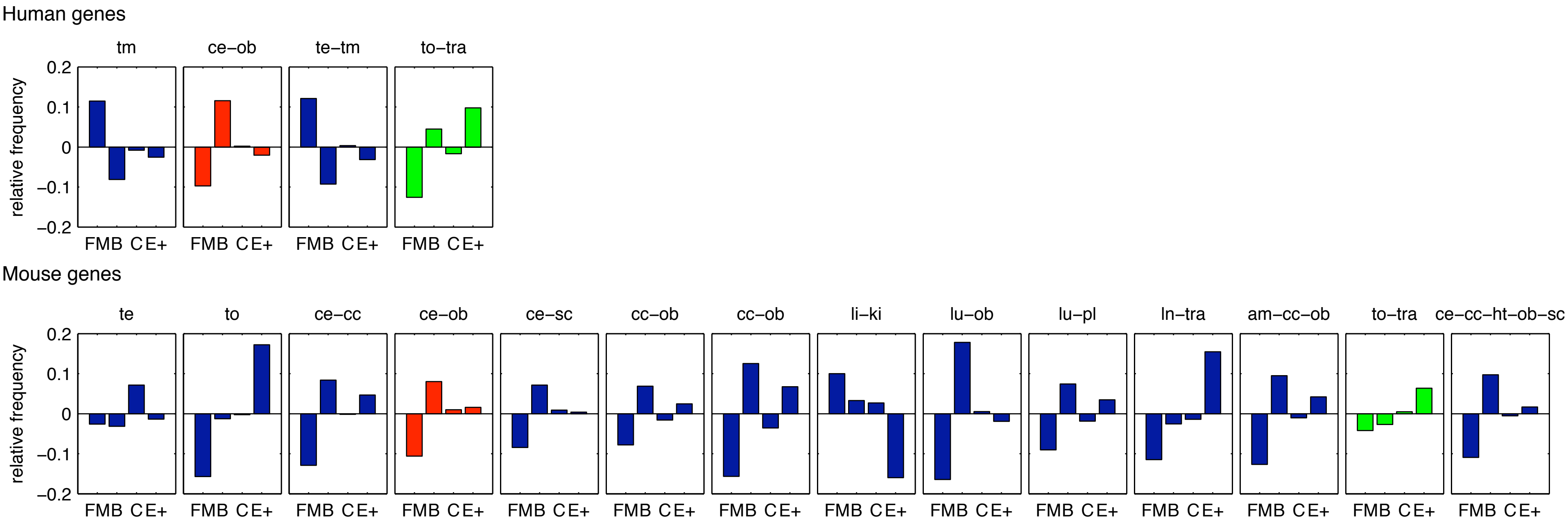

Table S1 - List of co-modules enriched with duplicated genes

co-module (human)	p-value	co-module (mouse)	p-value
cerebellum_cerebralcortex	3.97E-26	cerebralcortex_olfactorybulb	2.16E-20
cerebralcortex	3.79E-18	lung_swimbladder_placenta	9.98E-19
amygdala_cerebellum_cerebralcortex	2.86E-16	amygdala_cerebralcortex_hypothalamus	2.78E-17
cerebralcortex_olfactorybulb	1.80E-14	lung_swimbladder_lymphnode_trachea	1.48E-16
amygdala_cerebralcortex_olfactorybulb	9.88E-13	lung_swimbladder_uterus	2.01E-16
amygdala_cerebralcortex_hypothalamus_olfactorybulb	3.84E-12	amygdala_cerebralcortex_hypothalamus_olfactorybulb_spinalcord	1.05E-15
amygdala_cerebellum	4.89E-12	cerebralcortex_olfactorybulb	5.42E-15
lung_swimbladder_olfactorybulb	1.90E-11	amygdala_cerebellum_cerebralcortex_hypothalamus_olfactorybulb_spinalcord	2.14E-14
cerebellum_olfactorybulb	1.75E-10	amygdala_cerebralcortex_olfactorybulb	3.01E-14
amygdala_cerebralcortex_hypothalamus	1.04E-09	amygdala_cerebralcortex_hypothalamus_olfactorybulb	3.53E-14
cerebralcortex_olfactorybulb	3.44E-09	amygdala_cerebellum_cerebralcortex_hypothalamus_olfactorybulb_spinalcord	3.71E-14
tongue_trachea	3.61E-09	amygdala_cerebellum_cerebralcortex_hypothalamus_olfactorybulb_spinalcord	3.90E-14
lung_swimbladder_trachea	2.06E-08	amygdala_cerebralcortex_hypothalamus_olfactorybulb	6.87E-14
amygdala_cerebralcortex	2.28E-08	tongue_uterus	7.36E-14
amygdala_cerebralcortex_olfactorybulb	2.84E-08	cerebralcortex_spinalcord	1.25E-12
amygdala_cerebralcortex_hypothalamus_olfactorybulb_spinalcord	8.33E-08	cerebellum_cerebralcortex	8.25E-12
amygdala_cerebralcortex	1.39E-07	lung_swimbladder_olfactorybulb	1.53E-11
cerebellum_cerebralcortex_hypothalamus_olfactorybulb_spinalcord	1.68E-07	cerebellum_spinalcord	2.61E-11
adrenalgland_interrenalgland_cerebralcortex	3.27E-07	cerebellum_cerebralcortex_hypothalamus_olfactorybulb_spinalcord	4.93E-11
cerebellum	4.10E-07	amygdala_hypothalamus	6.08E-11
uterus	4.62E-07	amygdala_cerebralcortex_olfactorybulb	3.91E-10
prostate_trachea	2.11E-06	cerebellum_olfactorybulb	1.42E-09
adrenalgland_interrenalgland_trachea	5.40E-06	metanephros_olfactorybulb	1.21E-07
cerebellum_prostate	8.73E-06	placenta	3.44E-07
placenta	1.30E-05	tongue	3.95E-07
amygdala_hypothalamus	1.37E-05	lung_swimbladder	7.99E-07
ovary_trachea	4.17E-05	liver_spinalcord	2.17E-06
amygdala_hypothalamus_spinalcord	5.07E-05	olfactorybulb_spinalcord	2.74E-06
skeletalmuscle	5.21E-05	lymphnode	3.83E-06
spinalcord	8.33E-05	amygdala_cerebellum	4.18E-06
lymphnode_trachea	8.56E-05	amygdala_cerebralcortex_lung_swimbladder	4.57E-06
prostate_uterus	0.00011002	placenta_prostate	1.01E-05
olfactorybulb	0.00011907	cerebralcortex_hypothalamus_spinalcord	1.85E-05
trachea	0.00014606	uterus	2.15E-05
olfactorybulb_ovary	0.00022587	olfactorybulb	2.25E-05
ovary_uterus	0.00024082	lymphnode_uterus	3.29E-05
cerebellum_cerebralcortex_hypothalamus_spinalcord	0.00032101	amygdala_cerebralcortex	6.70E-05
amygdala	0.00037179	hypophysis_placenta	0.00012269
metanephros_olfactorybulb	0.0004573	amygdala_cerebralcortex	0.00014004
adrenalgland_interrenalgland_tongue	0.00046127	liver_metanephros	0.00015745
		cerebralcortex_hypothalamus	0.00016798
		tongue_trachea	0.00018484
		liver	0.00023893
		amygdala	0.00025849
		amygdala_cerebellum_cerebralcortex	0.00026879
		prostate_uterus	0.00028996
		lymphnode_thymus_trachea	0.0003495
