# Supplementary figures and images for: Comparative modular analysis of gene expression in vertebrate organs
Source: BMC Genomics. 2012 Mar 29;13:124. doi: 10.1186/1471-2164-13-124 (PMC3359279; doi:10.1186/1471-2164-13-124)

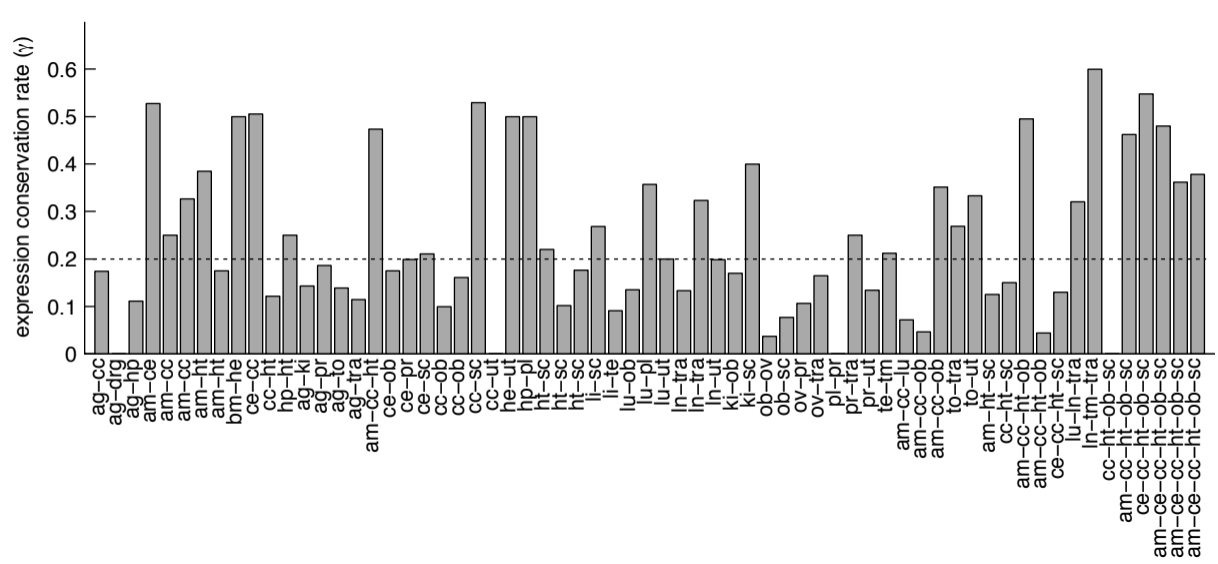

Supplement: Additional file 5 — Expression conservation rate (γ) for other system-specific co-modules. The median γ for all co-modules is marked with dotted line. Abbreviations for organ names: ag - adrenal gland; am - amygdala; bm - bone marrow; ce - cerebellum; cc - cerebral cortex; drg - dorsal root ganglion; he - heart; hp - hypophysis; ht - hypothalamus; li - liver; lu - lung; ln - lymph node; ki - kidney; ob - olfactory bulb; ov - ovary; pl - placenta; pr - prostate; sc - spinal cord; te - testis; tm - thymus; to -tongue; tra - trachea; ut - uterus. [file 1471-2164-13-124-S5.PDF]
